# The impact of high-intensity interval exercise training on NK-cell function and circulating myokines for breast cancer prevention among women at high risk for breast cancer

**DOI:** 10.1007/s10549-021-06111-z

**Published:** 2021-02-08

**Authors:** Adriana M. Coletta, Nadia H. Agha, Forrest L. Baker, Grace M. Niemiro, Preteesh L. Mylabathula, Abenaa M. Brewster, Therese B. Bevers, Enrique Fuentes-Mattei, Karen Basen-Engquist, Susan C. Gilchrist, Richard J. Simpson

**Affiliations:** 1 Department of Health and Kinesiology, The University of Utah, Salt Lake City, UT USA; 2 Cancer Control and Population Sciences Program, Huntsman Cancer Institute, Salt Lake City, UT USA; 3 Department of Health and Human Performance, The University of Houston, Houston, TX USA; 4 Department of Pediatrics, The University of Arizona, Tucson, AZ USA; 5 Department of Nutritional Sciences, The University of Arizona, Tucson, AZ USA; 6 Department of Clinical Cancer Prevention, The University of Texas MD Anderson Cancer Center, Houston, TX USA; 7 Department of Radiation Oncology Clinical Research, The University of Texas MD Anderson Cancer Center, Houston, TX USA; 8 Department of Behavioral Science, The University of Texas MD Anderson Cancer Center, Houston, TX USA; 9 Department of Cardiology, The University of Texas MD Anderson Cancer Center, Houston, TX USA; 10 Department of Immunobiology, The University of Arizona, Tucson, AZ USA; 11 The University of Arizona Cancer Center, Tucson, AZ USA; 12 Huntsman Cancer Institute at the University of Utah, 2000 Circle of Hope Drive, Research South Building Room 4747, Salt Lake City, UT 84112 USA

**Keywords:** Exercise immunology, Cytokines, Breast cancer prevention

## Abstract

**Purpose:**

Preclinical evidence suggests that natural killer cell (NK-cell) function and myokines facilitate the protective effects of exercise for breast cancer prevention. Since higher-intensity exercise acutely promotes greater mobilization and larger changes in NK-cell cytotoxicity than lower-intensity, high-intensity interval training (HIIT) might offer increased immune protection compared to moderate-intensity continuous-training (MICT). This study compared a 12-week HIIT program to a 12-week MICT program and usual care on changes in resting NK-cell function and circulating myokines among women at high risk for breast cancer.

**Methods:**

Thirty-three women were randomized to HIIT, MICT, or usual care, for a supervised exercise intervention. Blood was collected at baseline and end-of-study. The cytotoxic activity of CD3−/CD56+ NK-cells against the K562 target cell line in vitro was determined by flow cytometry. Circulating myokines (IL-15, IL-6, irisin, OSM, osteonectin, IL-7) were assessed with luminex multiplex assays and ELISA. One-way ANOVA and paired sample t-tests assessed between- and within-group differences, respectively. Pearson correlation coefficients determined relationships between baseline fitness and change variables.

**Results:**

Significant differences were not observed between groups for change in NK-cell function or circulating myokines (*p* > 0.05). Significant correlations were only observed for baseline peak aerobic capacity (ml/kg/min) and change in NK-cell-specific lysis (*r* = − 0.43, *p* = 0.02) and hemacytotoxicity for the total sample (*r* = − 0.46, *p* = 0.01).

**Conclusion:**

Our findings suggest that exercise intensity may not significantly impact change in resting NK-cell function and circulating myokines among women at high risk for breast cancer. Structured exercise training may have a larger impact on NK-cell function in those with lower levels of cardiorespiratory fitness.

**Clinical trial registration:** NCT02923401; Registered on October 4, 2016

## Introduction

High-intensity interval exercise training (HIIT), compared to traditional moderate-intensity continuous aerobic exercise training (MICT), promotes favorable changes in markers of health, notably cardiorespiratory fitness (CRF), cardiac function, blood lipids, oxidative stress, inflammation, insulin sensitivity, and quality of life, among apparently healthy and clinical populations [1–4]. HIIT requires less time to achieve the same exercise dose (i.e., metabolic equivalent hours) compared to MICT, which is advantageous because time is the biggest reported barrier to engaging in regular exercise among both apparently healthy and clinical populations [5]. In addition to the health benefits and time efficiency of HIIT, high-intensity exercise exhibits utility in the context of breast cancer prevention, such that greater than 6 h per week of vigorous-intensity physical activity over a lifetime is associated with a 23% reduction in breast cancer risk [6]. Breast cancer is the most common cancer among women worldwide, and the majority of cases occur in postmenopausal women [7, 8]. In addition to menopausal status, obesity, a history of non-invasive breast cancer (i.e., ductal carcinoma in situ or lobular carcinoma in situ), and an elevated Gail Model risk score, significantly increase a woman’s risk of developing breast cancer [9, 10].

There are multiple proposed mechanisms that underpin the association between exercise and breast cancer risk. Of particular interest to HIIT is the interplay of mobilization, redistribution, and effector functions of lymphocytes, particularly natural killer cells (NK-cells), and muscle-derived cytokines (e.g., ‘myokines’) released from skeletal muscle during contraction [11–13]. A recent review by Duggal et al. [14] suggests an association between exercise-induced myokine release and subsequent changes in the composition and function of the peripheral leukocyte compartment. Specific to breast cancer, in vitro evidence demonstrates that the myokine irisin suppresses malignant breast epithelial cell number and migration [15], and oncostatin M (OSM) reduces cellular proliferation and increases apoptosis [11]. Evidence from human studies exhibit an acute increase in irisin following exercise, and CRF is a strong predictor of the magnitude of change of irisin expression after acute exercise [16]. Observational evidence suggests a protective effect between elevated irisin levels at rest and risk of invasive breast cancer [17].

In coordination with immune function, IL-15 facilitates the expansion and maintenance of NK-cells and effector T-cells [18], while IL-6 has been shown to promote trafficking and tumor infiltration of exercise-mobilized NK-cells in several murine cancer models [19]. The cytotoxic activity of NK-cells against various cancer cell lines has been found to increase in response to both acute and chronic exercise, which has been attributed to phenotypic shifts in NK-cells and their frequent mobilization and redistribution with each bout of exercise [20, 21]. Because higher-intensity exercise acutely promotes greater mobilization and larger change in NK-cell cytotoxicity than lower-intensity exercise [20], HIIT might offer increased levels of immune protection in postmenopausal women with obesity at high risk of breast cancer compared to MICT. Moreover, a recent case-cohort study observed an inverse association between breast cancer risk and higher resting levels of NK-cells among postmenopausal women, although the association was not statistically significant [22].

We previously compared 12 weeks of HIIT, to 12 weeks of MICT, and usual care (non-exercise control) among postmenopausal women with obesity considered at high risk of breast cancer [2]. We observed significantly greater improvements in CRF variables with HIIT compared to MICT and usual care (UC) [2]. The purpose of this study was to determine the impact of this 12-week HIIT program compared with MICT and usual care on changes in resting NK-cell function and circulating myokines among women at high risk for breast cancer.

## Materials and methods

### Study design, participants, recruitment, and intervention procedures

This was a randomized controlled trial, where randomization was stratified by body mass index (BMI). Participants were randomized to HIIT, MICT, or UC for a 12-week intervention. This investigation was approved by the University of Texas MD Anderson Cancer Center Institutional Review Board. Details regarding eligibility criteria, recruitment, and intervention procedures are reported elsewhere [2].

Briefly, eligible participants consisted of postmenopausal women with overweight or obesity status, as defined by body mass index (≥ 25 kg/m^2^), who were considered at heightened risk of developing breast cancer due to an elevated Gail 5-year risk score (> 1.66%), lifetime risk score (> 20%), history of ductal or lobular atypia, or history of ductal or lobular carcinoma in situ (non-invasive breast cancer). Participants were recruited from the University of Texas MD Anderson Cancer Center, Clinical Cancer Prevention Center.

Participants assigned to HIIT and MICT presented at the Cancer Prevention Center three times per week and completed their supervised exercise sessions on a treadmill. HIIT consisted of an aerobic interval training protocol. Following a 5-min warm-up at 50–70% peak heart rate (HR), four 4-min high-intensity intervals were completed at 90–100% peak HR, with each high-intensity interval followed by a 3-min active recovery interval at 50–70% peak HR. The HIIT workout was 33 min in length. The MICT workout was 41 min in length, completed at 60–70% peak HR. Participants assigned to UC received educational material related to healthful diet and exercise habits at baseline, and monthly phone calls by study personnel pertaining to their healthy lifestyle goals.

### Assessment procedures

Details regarding assessment procedures are reported elsewhere [2]. Briefly, assessment sessions were conducted at baseline, 6 weeks and upon completion of the 12-week intervention (end-of-study). Relevant to the present investigation, cardiopulmonary exercise testing was conducted at baseline, 6 weeks and end-of-study, utilizing the modified Balke protocol [23], to measure CRF variables. Peak HR measured from the cardiopulmonary exercise testing was used to prescribe individualized HR training zones. Additionally, fasting whole blood was collected at baseline and end-of-study using standard phlebotomy procedures. The median and average number of days between last workout and end-of-study assessment blood draw were 3 and 5 days, respectively. Upon completion of the last training session and prior to the participant’s end-of-study assessment, participants were instructed to continue their normal diet and lifestyle.

### NK-cell function analysis

At baseline and end-of-study, fasting whole blood was collected into one 8.5 mL ACD-containing collection tube, one 6 mL lithium heparin-containing collection tube, and one 2.5 mL EDTA-containing collecting tube. Peripheral blood mononuclear cells (PBMCs) were isolated from 8.5 mL whole blood by density gradient centrifugation (Histopaque-1077, Sigma-Aldrich). The HLA-deficient leukemia cell line K562 (ATCC: CCL-143) was maintained in a glutamine-enriched, 10% FBS and 1% penicillin streptomycin RPMI-1640 (Sigma-Aldrich) and used as target cells for the cytotoxicity assay. On the day of the NK-cell assay, K562 cells were stained with anti-CD71 FITC antibody (IgG1, Clone OKT9), washed, and re-suspended in media (10% FBS and 1% penicillin streptomycin RPMI-1640). Monocyte-depleted PBMCs were co-cultured with CD71-labeled K562 cells at 0:1 (spontaneous cell death), 1:1, 5:1, 10:1, and 20:1 effector (PBMCs): target (K562) cell ratios for 4 h in a humidified CO2 incubator at 37 °C. The same number of PBMCS and target cells were used for baseline and end-of-study samples. NK-cells were quantified as a percentage of isolated PBMCs [24]. NK-cell cytotoxicity was determined by flow cytometry as previously described [25]. Hemacytotoxicity was defined as the specific lysis (% total lysis − % spontaneous death) of 10,000 target cells from 50 uL of whole blood. Overall, the following variables were used to assess NK-cell function: NK-cells as percent of lymphocytes, NK-cell counts (cell/uL), hemacytotoxicity, lytic index, and specific lysis.

### Myokine analysis

#### Irisin, Oncostatin M (OSM), IL-15, IL-6, Osteonectin

Fasting whole blood was collected into one, 6 mL EDTA-containing collection tube at baseline and end-of-study. Once blood was collected, the EDTA tube was centrifuged at 1000 g for 10 min and plasma was then aliquoted in 1.5 mL microcentrifuge tubes and stored at − 80 °C until completion of the trial for batch analysis. Upon completion of the trial, plasma samples were analyzed via a customized, Luminex® Human Myokine Magnetic Bead Panel (HMYOMAG-56 K, Millipore Corporation, Billerica MA) using a Luminex 200™ System with Luminex YX Platform™ software. In brief, 35 μL of plasma were diluted 1:2 with 35 μL of Assay Buffer and 25 µL of diluted sample were run in duplicates following the kit-optimized protocol recommended by the company (Millipore Corporation, Billerica MA). The following myokines were included in the panel: irisin, OSM, IL-15, IL-6, and osteonectin. The intra-assay coefficient of variation for these panels are < 10%. Some myokines within the luminex multiple assay resulted in undetectable levels. Undetectable levels were defined as follows: IL-15 < 2.44 pg/mL, IL-6 < 0.59 pg/mL, irisin < 244.14 pg/mL, and osteonectin < 7.32 pg/mL. For IL-15, three samples included undetectable levels in the HIIT, MICT, and usual care. For IL-6 only one sample included undetectable levels for MICT. For irisin, one sample in MICT and five in usual care contained undetectable levels. Finally, for osteonectin, only one sample in the usual care group contained undetectable levels.

#### IL-7

Plasma samples were analyzed in duplicate for IL-7 concentrations via ELISA (R&D Systems, Minneapolis, MN) following the manufacturer’s instructions. The lower level of detection of the kit used was less than 0.1 pg/mL. Samples were read using a SpectraMax M3 plate reader (Molecular Devices, LLC, San Jose CA) and analyzed on SoftMax Pro (v. 6.3, Molecular Devices, LLC, San Jose CA). The intra-assay coefficient of variation for this plate was 10.82%. All samples had detectable levels of IL-7 in plasma.

### Statistical analysis

We analyzed our data based on the intention-to-treat principle. One-way analysis of variance (ANOVA) was used to assess differences between groups at baseline and end-of-study, and for changes from baseline (end-of-study value – baseline value) for NK-cell function and myokine variables. General linear model univariate analysis of covariance was used to assess within- and between-group differences in changes in NK-cell function and myokine variables, controlling for baseline age and BMI. Paired sample t-tests were used to assess within-group differences from baseline to end-of-study. Pearson correlation coefficients were assessed to determine the relationship between: (1) baseline peak aerobic capacity (ml/kg/min) and change in both NK-cell function and myokines for the total sample and by group, (2) change in peak aerobic capacity (ml/kg/min) and change in NK-cell function and myokines, (3) change in NK-cell function and change in myokines. In addition, Fisher’s r to z transformation was completed, using a two-tailed test of significance, to test for potential differences in correlations between groups for the comparison of baseline peak aerobic capacity and change in NK-cell function and myokines. Data were analyzed with IBM® SPSS® Version 26 software (IBM Corp., Armonk, NY, USA).

## Results

### Participant characteristics

At baseline, there were no significant differences between groups among the following variables: age, body mass index, cardiorespiratory fitness, race, ethnicity, status of breast cancer risk (i.e., history of ductal or lobular carcinoma in situ, atypia, elevated risk scores) [2]. Thirty-three participants completed the trial. Twelve participants who completed the intervention were randomized to HIIT, 10 completers to MICT, and 11 completers to UC. Table 1 presents baseline characteristics of the 33 participants who completed the intervention.

**Table 1:** Baseline characteristics of completers

	HIIT	MICT	UC
	Mean (SD)	Mean (SD)	Mean (SD)
Age (years)	64.4 (6.2)	66.1 (13.6)	62.6 (7.0)
BMI (Kg/m^2^)	32.3 (8.6)	32.0 (5.6)	29.9 (2.5)
VO2 peak (ml/kg/min)	18.8 (3.2)	20.8 (3.5)	19.4 (3.8)

	*N* (%)	*N* (%)	*N* (%)
Elevated Gail	10 (84%)	9 (90%)	8 (73%)
DCIS	1 (8%)	0	2 (18%)
LCIS	1 (8%)	0	0
Atypia	0	1 (10%)	1 (9%)
Gail Risk Score (*n* = 27)	3.25 (1.90)	2.68 (0.74)	4.31 (4.31)

### NK-cell function

Table 2 presents group means and statistics for between-group differences for NK-cell function variables at baseline, end-of-study, and change from baseline in the unadjusted model. Specific lysis from the 20:1 E:T ratio is reported. Overall, significant differences were not observed within or between groups for NK-cell function variables in unadjusted and adjusted models.

**Table 2:** Differences between groups in NK-cell function

		*N*	Baseline	*p*-level	End-of-Study	*p*-level	Change from Baseline	*p*-level
NK-Cells (%Lymphocytes)	HIIT	12	9.56 ± 5.04	0.70	10.50 ± 3.60	0.66	0.94 ± 4.99	0.98
	MICT	10	8.24 ± 5.27		8.80 ± 7.31		0.56 ± 4.70	
	UC	11	7.95 ± 4.17		8.66 ± 4.86		0.71 ± 3.57	
NK-Cell Counts (cell/uL)	HIIT	12	175.18 ± 88.68	0.48	188.15 ± 54.44	0.50	12.98 ± 87.97	0.99
	MICT	10	134.44 ± 75.23		146.30 ± 94.99		11.86 ± 79.28	
	UC	11	145.12 ± 78.90		161.52 ± 99.09		16.40 ± 54.04	
Hemacytotoxicity (#dead cells/50 uL blood)	HIIT	12	20.60 ± 10.20	0.70	20.08 ± 12.96	0.80	− 0.52 ± 10.04	0.89
	MICT	10	17.84 ± 10.68		16.87 ± 9.81		− 0.97 ± 5.28	
	UC	11	17.45 ± 8.38		18.11 ± 11.18		0.65 ± 7.95	
Lytic Index (#dead target cells/NK-cell)	HIIT	12	412.18 ± 239.62	0.89	344.40 ± 226.41	0.90	− 67.78 ± 237.86	0.43
	MICT	10	350.34 ± 270.56		393.46 ± 377.58		43.12 ± 205.38	
	UC	11	403.25 ± 403.06		341.32 ± 268.03		− 61.93 ± 205.40	
Specific Lysis (%killed target cells/10,000 NK-cells)	HIIT	12	23.71 ± 13.19	0.75	27.58 ± 15.41	0.84	3.87 ± 13.11	0.24
	MICT	10	28.41 ± 20.90		24.76 ± 13.72		− 3.65 ± 11.78	
	UC	11	24.12 ± 12.27		28.63 ± 17.07		4.51 ± 10.90	

**Fig. 1:**
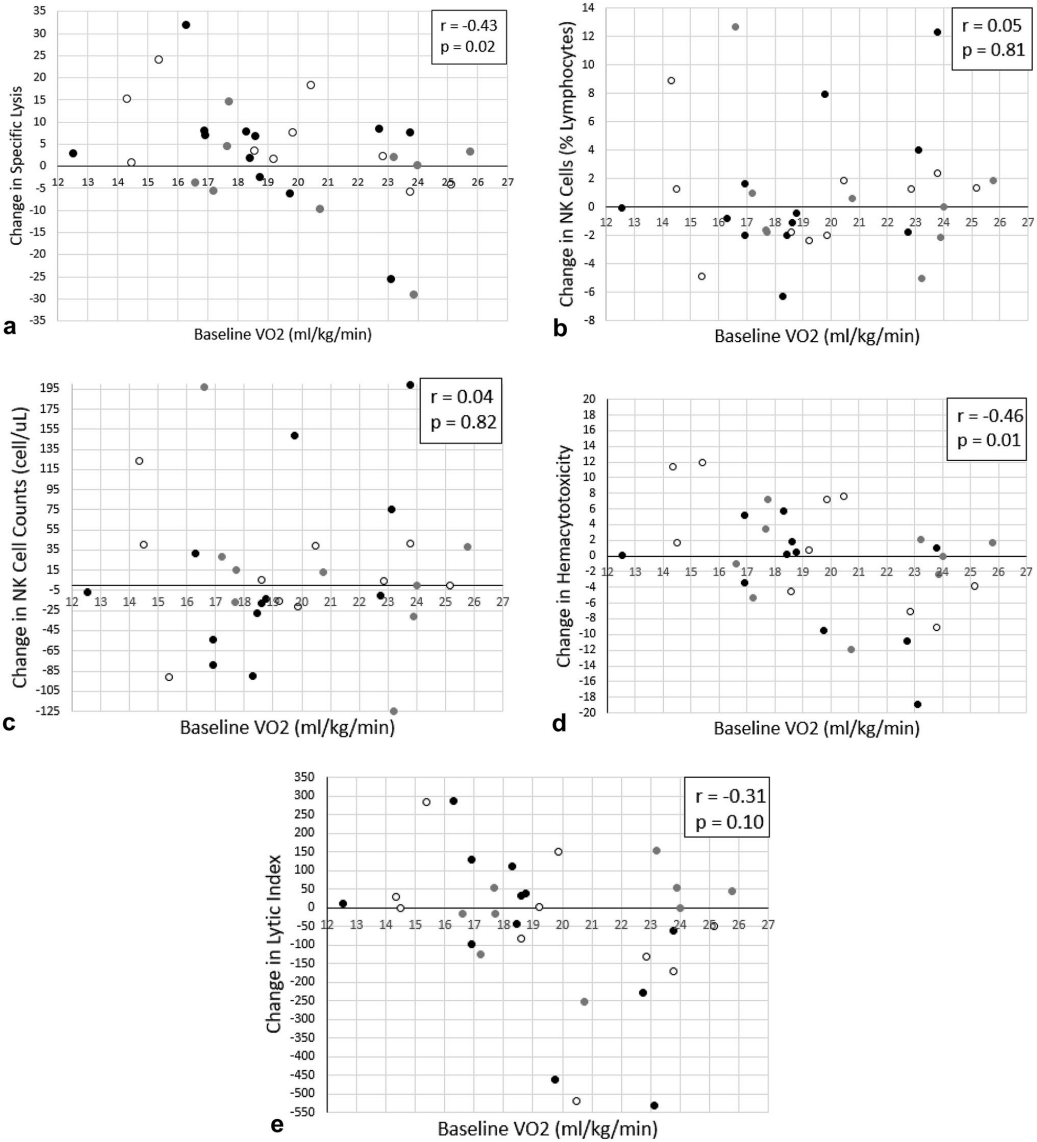
Correlation between baseline peak aerobic capacity (ml/kg/min) and change in markers of NK-Cell function for the total sample. **a** Specific lysis, **b** percent lymphocytes, **c **NK-cell counts, **d** hemacytotoxicity, **e** Lytic Index. *HIIT* black circles, *MICT* gray circles, *UC* white circles

A significant correlation was observed for baseline peak aerobic capacity (ml/kg/min) and change in a measure of NK-cell cytotoxicity, specific lysis, in all participants, but not by group (HIIT: *r* = − 0.39, z = 0.41, *p* = 0.21; MICT: *r* = − 0.27, *z* = 0.28, *p* = 0.49; UC: *r* = − 0.59, *z* = 0.68, *p* = 0.07). A significant correlation was also observed for baseline peak aerobic capacity (ml/kg/min) and a marker of NK-cell function, hemacytotoxicity, for the total sample (*r* = − 0.46, *p* = 0.01, *n* = 31) and in UC (*r* = − 0.70, *z* = 0.87, *p* = 0.02), but not in HIIT (*r* = − 0.54, *z* = 0.60, *p* = 0.07) or MICT (*r* = − 0.02, *z* = 0.02, *p* = 0.96). Significant correlations were not observed for the total sample or by group for NK-cells as percent lymphocyte (*r* = 0.05, *p* = 0.81; HIIT: *r* = 0.49, *z* = 0.54, *p* = 0.11; MICT: *r* = − 0.40, *z* = 0.42, *p* = 0.29; UC: *r* = − 0.05, *z* = 0.05, *p* = 0.90), NK-cell counts (*r* = 0.04, *p* = 0.82; HIIT: *r* = 0.54, *z* = 0.60, *p* = 0.07; MICT: *r* = − 0.47, *z* = 0.51, *p* = 0.20; UC: *r* = − 0.12, *z* = 0.12, *p* = 0.73), or lytic index (*r* = − 0.31, *p* = 0.10; HIIT: *r* = − 0.57, *z* = 0.65, *p* = 0.05; MICT: *r* = 0.35, *z* = 0.37, *p* = 0.36; UC: *r* = − 0.43, *z* = 0.46, *p* = 0.21) (Fig. 1).

Based on the z-score differences for each group, significant differences in correlations of baseline peak aerobic capacity and change in markers of NK-cell function between groups were not significant (*p* > 0.05; specific lysis: HIIT-vs-MICT = 0.27, MICT-vs-UC = − 0.77, HIIT-vs-UC = − 0.55; hemacytotoxicity: HIIT-vs-MICT = 1.16, MICT-vs-UC = − 1.64, HIIT-vs-UC = − 0.54; percent lymphocyte: HIIT-vs-MICT = 0.22, MICT-vs-UC = 0.72, HIIT-vs-UC = 1.00; NK-cell count: HIIT-vs-MICT = 0.19, MICT-vs-UC = 0.75, HIIT-vs-UC = 1.00; lytic index: HIIT-vs-MICT = 0.56, MICT-vs-UC = − 0.18, HIIT-vs-UC = 0.39). Significant correlations were not observed between change in peak aerobic capacity and change in NK-cell function.

Correlation analyses between change in markers of NK-cell function and change in myokines by total sample reveal a significant relationship between change in irisin and specific lysis (*r* = 0.42, *p* = 0.04, *n* = 25) and change in IL-6 and lytic index (*r* = 0.41, *p* = 0.02, *n* = 25). By group, HIIT (*n* = 12) revealed significant correlations between change in IL-7 and change in both percent lymphocytes (*r* = 0.72, *p* = 0.009), and NK-cell counts (*r* = − 0.66, *p* = 0.02), change in IL-6 and change in hemacytotoxicity (*r* = 0.61, *p* = 0.03), lytic index (*r* = 0.66, *p* = 0.02), and specific lysis (*r* = 0.81, *p* = 0.001). MICT only revealed significant correlations between change in IL-7 and change in percent lymphocytes (*r* = 0.91, *p* < 0.001) and NK-cell counts (*r* = 0.83, *p* = 0.003). UC revealed significant correlations between change in IL-7 and change in both NK-cell counts (*r* = 0.62, *p* = 0.04) and lytic index (*r* = 0.68, *p* = 0.02). No other significant correlations were observed.

#### Myokines

Table 3 presents group means and statistics for between-group differences for myokines at baseline, end-of-study, and change from baseline in the unadjusted model. Significant differences between groups were not observed at baseline and end-of-study (*p* > 0.05). Change from baseline was not significantly different between groups in the unadjusted and adjusted model. Within-group differences were only observed in usual care for IL-6 in the unadjusted model, such that participants exhibited a significant reduction in IL-6 from baseline to end-of-study (− 1.34 ± 1.74, *p* = 0.03). No other significant within-group differences were observed for myokines in the unadjusted and adjusted model.

**Table 3:** Differences between groups in myokines

		*N*	Baseline	*p*-level	End-of-Study	*p*-level	Change from Baseline	*p*-level
IL-15 (pg/mL)	HIIT	9	5.51 ± 6.06	0.14	5.01 ± 5.10	0.20	− 0.51 ± 2.78	0.17
	MICT	7	5.87 ± 4.74		6.53 ± 5.44		0.66 ± 0.97	
	UC	8	13.89 ± 13.77		12.02 ± 11.33		− 1.87 ± 3.02	
IL-6 (pg/mL)	HIIT	12	7.07 ± 5.61	0.68	6.14 ± 5.29	0.72	− 0.93 ± 4.16	0.91
	MICT	9	5.16 ± 4.60		4.37 ± 3.53		− 0.79 ± 2.10	
	UC	11	7.51 ± 7.73		6.17 ± 7.01		− 1.34 ± 1.74	
Irisin (pg/mL)	HIIT	10	323.35 ± 344.39	0.21	356.69 ± 339.35	0.25	33.34 ± 299.39	0.60
	MICT	9	464.68 ± 469.68		375.63 ± 353.65		− 89.04 ± 293.10	
	UC	6	795.59 ± 735.80		722.03 ± 667.03		− 73.56 ± 202.08	
OSM (pg/mL)	HIIT	12	3.87 ± 1.90	0.55	3.70 ± 1.89	0.35	− 0.17 ± 1.45	0.90
	MICT	10	5.18 ± 5.05		5.37 ± 5.59		0.19 ± 1.06	
	UC	11	6.15 ± 6.77		6.27 ± 4.63		0.12 ± 2.85	
Osteonectin (pg/mL)	HIIT	12	253.10 ± 125.95	0.44	264.94 ± 171.58	0.90	11.85 ± 77.07	0.30
	MICT	10	294.52 ± 107.93		269.26 ± 99.30		− 25.25 ± 45.83	
	UC	10	235.45 ± 65.97		245.69 ± 59.44		10.24 ± 46.60	
IL-7 (pg/mL)	HIIT	12	2.57 ± 4.33	0.37	1.00 ± 0.71	0.15	− 1.57 ± 4.21	0.10
	MICT	10	1.44 ± 1.08		2.39 ± 2.52		0.95 ± 2.00	
	UC	11	0.98 ± 0.71		1.47 ± 1.33		0.49 ± 1.37	

Regarding correlation analyses, baseline peak aerobic capacity (ml/kg/min) was not associated with change in IL-15 (*r* = − 0.13, *p* = 0.55, *n* = 23), IL-6 (*r* = − 0.28, *p* = 0.14, *n* = 30), IL-7 (*r* = − 0.26, *p* = 0.15, *n* = 31), irisin (*r* = − 0.34, *p* = 0.11, *n* = 23), OSM (*r* = − 0.13, *p* = 0.48, *n* = 31), or osteonectin (*r* = − 0.04, *p* = 0.85, *n* = 31) for the total sample. Significant associations were also not observed by group for IL-15 (HIIT: *r* = − 0.27, *z* = 0.26, *p* = 0.49; MICT: *r* = 0.37, *z* = 0.35, *p* = 0.47; UC: *r* = − 0.28, *z* = 0.28, *p* = 0.51), IL-6 (HIIT: *r* = − 0.50, *z* = 0.55, *p* = 0.10; MICT: *r* = 0.05, *z* = 0.05, *p* = 0.91; UC: *r* = − 0.25, *z* = 0.26, *p* = 0.49), IL-7 (HIIT: *r* = − 0.47, *z* = 0.51, *p* = 0.17; MICT: *r* = − 0.26, *z* = 0.26, *p* = 0.49; UC: *r* = − 0.50, *z* = 0.55, *p* = 0.14), irisin (HIIT: *r* = − 0.28, *z* = 0.29, *p* = 0.44; MICT: *r* = − 0.37, *z* = 0.39, *p* = 0.37; UC: *r* = − 0.39, *z* = 0.41, *p* = 0.52), OSM (HIIT: -0.38, *z* = 0.40, *p* = 0.22; MICT: *r* = 0.43, *z* = 0.46, *p* = 0.24; UC: *r* = − 0.23, *z* = 0.23, *p* = 0.53), or osteonectin (HIIT: 0.21, *z* = 0.21, *p* = 0.51; MICT: *r* = 0.26, *z* = 0.27, *p* = 0.50; UC: *r* = − 0.50, *z* = 0.55, *p* = 0.15).

Based on the z-score differences for each group, significant differences in correlations of baseline peak aerobic capacity and change in myokines between groups were not significant (*p* > 0.05; IL-15: HIIT-vs-MICT = − 0.14, MICT-vs-UC = 0.10, HIIT-vs-UC = − 0.03; IL-6: HIIT-vs-MICT = 0.95, MICT-vs-UC = − 0.38, HIIT-vs-UC = 0.60; IL-7: HIIT-vs-MICT = 0.48, MICT-vs-UC = − 0.53, HIIT-vs-UC = − 0.08; irisin: HIIT-vs-MICT = − 0.18, MICT-vs-UC = − 0.03, HIIT-vs-UC: -0.18; OSM: HIIT-vs-MICT = − 0.12, MICT-vs-UC = 0.44, HIIT-vs-UC = 0.34; osteonectin: HIIT-vs-MICT = − 0.10, MICT-vs-UC = − 0.53, HIIT-vs-UC = − 0.67). Significant correlations were not observed between change in peak aerobic capacity and change in myokines.

## Discussion

We aimed to compare the impact of a 12-week high-intensity interval training (HIIT) program to a 12-week moderate-intensity continuous aerobic training (MICT) program on changes in resting NK-cell function and circulating myokines among postmenopausal women with obesity at heightened risk for breast cancer. We previously reported that this trial demonstrated low attrition rates, and high adherence and compliance rates (adherence: 90% in HIIT and 89% in MICT; compliance: 100% to HIIT and MICT protocols), and greater improvements in cardiorespiratory fitness (CRF) after 12 weeks of HIIT compared to MICT and usual care [2], yet in the present investigation, we did not observe significant changes in resting NK-cell function following 12 weeks of training. This suggests that exercise training, regardless of intensity, may not influence the ability of NK-cells to kill tumor target cells in vitro. While we observed an increase in NK-cell counts across all groups, these findings are consistent with a recent 12-week exercise intervention conducted among postmenopausal women with obesity (but not at heightened risk for breast cancer) that compared moderate-intensity continuous aerobic exercise to resistance training and a non-exercise control [26].

Additionally, our findings are similar to a 12-month moderate-intensity continuous aerobic exercise intervention conducted among postmenopausal women with obesity, such that no effects on change in natural killer cell cytotoxicity were observed [27]. Moreover, a recent pilot study in breast cancer patients found no effect of a 12-week resistance exercise training intervention on the gene expression profile of peripheral blood NK-cells [28]. Taken together, these findings suggest that chronic exercise, regardless of intensity, may not impact changes in resting (e.g., > 24 h since the last exercise bout) NK-cell function in vitro or NK-cell gene expression. However, because exercise training has been shown to increase NK-cell trafficking and infiltration of tumors in vivo [19], it is possible that other aspects of NK-cell function are improved with exercise training such as mobilization, tissue migration, tumor infiltration, and the ability to secrete and be activated by various cytokines [18].

Since fitness (e.g., higher CRF) is associated with greater levels of NK-cell function [29–31] and a greater release of myokines into the circulation immediately following exercise [16], we explored the relationship between baseline fitness levels and changes in immune function and myokines following exercise training. We did not observe significant correlations between change in circulating myokines and baseline fitness for the total sample or by group, which suggests that baseline CRF may not impact the magnitude of change of resting levels of myokines after participating in a 12-week exercise program. We did, however, find a significant relationship between baseline CRF levels and the change in NK-cell function, indicating that a positive change in NK-cell function after an exercise training intervention might be more apparent in those with lower CRF at baseline. However, these findings should be interpreted with caution, due to our smaller sample size and exploratory nature of this analysis.

Regarding myokines, we assessed irisin, OSM, osteonectin, IL-6, IL-15, and IL-7 on account of evidence supporting the utility of these myokines in the context of cancer prevention and control [11, 15, 19, 32–34]. Additionally, IL-15 and IL-6 have known roles in activating NK-cell and T-cells during exercise [18] and ability to facilitate the immune system and promote anti-cancer effects in preclinical models [19, 33]. We did not observe significant differences between groups for changes in circulating myokines at rest after participation in a 12-week exercise training program. Among OSM, IL-15, IL-6, and IL-7 we observed either a small reduction in levels from baseline or negligible increase within groups. Together, these findings suggest that exercise intensity may not influence the extent of change in these circulating myokines at rest.

While we did not observe statistically significant differences between or within groups for changes in plasma osteonectin or irisin, we observed a larger extent of change in both directions, such that HIIT was associated with an increase in change in resting levels, and MICT with a decrease. Our findings related to osteonectin are consistent with a previous investigation that observed an increase in resting mRNA expression of osteonectin in skeletal muscle after participation in a HIIT program [35]. For irisin, an increase after HIIT aligns with preclinical and human studies demonstrating an increase in gene expression of irisin (also referred to as fibronectin type III domain-containing protein 5 [FNDC5]) following high-intensity exercise specifically [36, 37]. Evidence in humans has also demonstrated an increase in irisin gene expression at rest for up to 20 days after high-intensity exercise [36]. We also observed a reduction in resting levels of irisin after MICT, which is consistent with a recent meta-analysis conducted by Qui et al. [38].

Interestingly, when comparing the correlations of change in NK-cell function and myokines, participation in HIIT was associated with the most number of correlations in changes in NK-cell function and myokines. These findings suggest the synergistic effects of high-intensity exercise on NK-cell function and myokines. These findings may serve as early evidence related to the underlying physiological mechanisms that associate vigorous-intensity exercise with reductions in breast cancer risk [6].

This is the first study to our knowledge to compare the chronic effects of different types of exercise intensities on changes in resting NK-cell function and circulating myokines among women at heightened risk of breast cancer, who are also postmenopausal with obesity. We consider this a strength of our trial. Other strengths include the supervised exercise intervention and measurement methods used to assess circulating myokines (luminex multiplex panel) and NK-cell function. However, our study is not without limitations. Specific to the myokine analysis, we consider the inconsistent timing for blood collection in relation to last workout across participants a limitation of our study. Additionally, the smaller sample size, use of K562 cell line and only one cell line for NK-cell analyses are also limitations to our findings.

Future studies assessing exercise-induced changes in resting NK-cell function in the context of breast cancer should utilize a broad range of breast cancer cell lines. Additionally, future work should consider assessment of other aspects of NK-cell function in vivo (e.g., mobilization and redistribution), and assessment of baseline CRF as it compares to changes in markers of immune function due to exercise. Future research assessing myokines specifically should consider consistent timing of blood collection in relation to completion of the exercise training program. Furthermore, findings from this exploratory analysis provide preliminary estimates that can be used in future studies for power calculation. In summary, our findings suggest that exercise intensity does not significantly impact resting in vitro NK-cell function and change in circulating myokines after completion of a 12-week exercise training program among postmenopausal women with obesity and at heightened risk of breast cancer.

## Data Availability

All data generated or analyzed during this study are available upon request to the corresponding author.
